# The Stress-Inducible Peroxidase *TSA2* Underlies a Conditionally Beneficial Chromosomal Duplication in *Saccharomyces cerevisiae*

**DOI:** 10.1534/g3.117.300069

**Published:** 2017-07-26

**Authors:** Robert A. Linder, John P. Greco, Fabian Seidl, Takeshi Matsui, Ian M. Ehrenreich

**Affiliations:** * Molecular and Computational Biology Section, Department of Biological Sciences, University of Southern California, Los Angeles, California 90089-2910; † Department of Ecology and Evolutionary Biology, School of Biological Sciences, University of California, Irvine, California 92697-2525

**Keywords:** aneuploidy chromosomal, duplication natural genetic variation oxidative stress yeast

## Abstract

Although chromosomal duplications are often deleterious, in some cases they enhance cells’ abilities to tolerate specific genetic or environmental challenges. Identifying the genes that confer these conditionally beneficial effects to particular chromosomal duplications can improve our understanding of the genetic and molecular mechanisms that enable certain aneuploidies to persist in cell populations and contribute to disease and evolution. Here, we perform a screen for spontaneous mutations that improve the tolerance of haploid *Saccharomyces cerevisiae* to hydrogen peroxide. Chromosome IV duplication is the most frequent mutation, as well as the only change in chromosomal copy number seen in the screen. Using a genetic mapping strategy that involves systematically deleting segments of a duplicated chromosome, we show that the chromosome IV’s duplication effect is largely due to the generation of a second copy of the stress-inducible cytoplasmic thioredoxin peroxidase *TSA2*. Our findings add to a growing body of literature that shows the conditionally beneficial effects of chromosomal duplication are typically mediated by a small number of genes that enhance tolerance to specific stresses when their copy numbers are increased.

Abnormalities in chromosomal copy number (or “aneuploidies”) often lead to cancer (Davoli *et al.* 2013; Potapova *et al.* 2013; Sheltzer 2013; Durrbaum and Storchova 2015, 2016; Laubert *et al.* 2015; Mohr *et al.* 2015; Nicholson and Cimini 2015; Pinto *et al.* 2015; Santaguida and Amon 2015), developmental defects (Ottesen *et al.* 2010; Gannon *et al.* 2011; Siegel and Amon 2012; Akasaka *et al.* 2013; Bose *et al.* 2015), premature aging (Andriani *et al.* 2016; Sunshine *et al.* 2016), and other health issues in humans. In the budding yeast *Saccharomyces cerevisiae*, aneuploidies also tend to be deleterious (Torres *et al.* 2007; Yona *et al.* 2012; Potapova *et al.* 2013; Dodgson *et al.* 2016; Sunshine *et al.* 2016). However, in some cases, these aneuploidies are conditionally beneficial, as they can enable yeast to tolerate specific loss-of-function mutations or environmental stresses (Selmecki *et al.* 2009, 2015; Pavelka *et al.* 2010; Chen *et al.* 2012a,b; Yona *et al.* 2012; Tan *et al.* 2013; Kaya *et al.* 2015; Liu *et al.* 2015; Meena *et al.* 2015; Sirr *et al.* 2015; Sunshine *et al.* 2015).

An important question regarding such conditionally beneficial aneuploidies is, do their effects tend to arise due to changes in the copy numbers of one or multiple genes on the aneuploid chromosome(s)? Several studies have attempted to address this question by identifying the specific genes underlying the conditionally beneficial effects of particular chromosomal duplications in budding yeast (Pavelka *et al.* 2010; Chen *et al.* 2012a; Kaya *et al.* 2015; Liu *et al.* 2015). For example, Kaya *et al.* found that chromosome XI duplication enabled *S. cerevisiae* strains lacking all eight thiol peroxidase genes to be nearly as tolerant to oxidative stress as a wild-type strain (Kaya *et al.* 2015). Two genes mediated the benefit of chromosome XI duplication: *CCP1*, a hydrogen peroxide scavenger that acts in the mitochondrial intermembrane space, and *UTH1*, a mitochondrial inner-membrane protein. In another case, Liu *et al.* showed that chromosome VIII duplication compensates for the absence of essential nuclear pore proteins by causing overexpression of a gene that regulates cell membrane fluidity (Liu *et al.* 2015). Furthermore, Chen *et al.* demonstrated that chromosome XV duplication confers resistance to the Hsp90 inhibitor radicicol by increasing the dosage of *PDR5* and *STI1*, which encode a multidrug pump and an Hsp90 cochaperone, respectively (Chen *et al.* 2012a). Lastly, Pavelka *et al.* showed that chromosome XIII duplication confers increased resistance to the DNA-damaging agent 4-NQO by increasing the dosage of *ATR1*, another multidrug pump (Pavelka *et al.* 2010). These studies suggest that the conditional benefits of aneuploidization are typically mediated by changes in the copy numbers of a small number of genes that allow cells to cope with specific stresses.

Here, we explore how aneuploidies can enable yeast to tolerate environmental stresses to a level beyond that achievable through genetic variation that segregates in natural populations (so-called “natural genetic variation”). We previously found that progeny produced by mating the laboratory strain BY4716 (BY), the vineyard isolate RM11-1a (RM), and the oak isolate YPS163 (YPS) show similar maximal hydrogen peroxide tolerances despite their genetic differences (see Supplemental Material, Figure S1 in File S1), suggesting the extent to which natural genetic variation can increase tolerance to this compound may be constrained (Linder *et al.* 2016). We attempted to overcome these limits by screening haploid segregants from the BY × RM, BY × YPS, and RM × YPS crosses for spontaneous mutations that increase hydrogen peroxide tolerance beyond the maximal levels seen in our past work. Specifically, we took the single most tolerant F_2_ segregant that we identified in each of the possible pairwise crosses of the three strains and used these three individuals as the progenitors in a screen for spontaneous mutations that enhance hydrogen peroxide resistance.

We obtained 37 mutants that show increased hydrogen peroxide tolerance relative to their respective progenitors. Using whole genome sequencing, we identified spontaneous mutations that may cause increased hydrogen peroxide tolerance in these mutants. Duplication of chromosome IV was the most frequent mutation, and the only aneuploidy detected in the screen. Consistent with chromosome IV duplication being conditionally beneficial, we found that chromosome IV aneuploids grow worse than their progenitors in the absence of hydrogen peroxide, and that the benefit of chromosome IV disomy occurs on agar plates but not in liquid media. Following on from these discoveries, we determined the genetic basis of this chromosomal duplication’s effect using chromosome- and gene-scale genetic engineering. Employing these techniques, we identified a single gene, the stress-inducible cytoplasmic thioredoxin peroxidase *TSA2*, which accounts for the majority of the effect of chromosome IV duplication on hydrogen peroxide tolerance. Our findings illustrate how aneuploidies can enable cells to surpass the levels of stress tolerance that are attainable through natural genetic variation and provide further support that the conditionally beneficial effects of aneuploidies tend to have a simple genetic basis.

## Materials and Methods

### Screen for increased hydrogen peroxide tolerance

Progenitor strains were produced during our past work on the BY × RM, BY × YPS, and RM × YPS crosses (Ehrenreich *et al.* 2012; Linder *et al.* 2016) and are described in more detail in Linder *et al.* (2016). Each progenitor strain was streaked onto yeast extract-peptone-dextrose (YPD) plates and incubated for 2 d at 30°. To maximize biological independence among mutations obtained from the screen, 24 different colonies per progenitor were individually inoculated into 800 μl of YPD broth. These cultures were outgrown for 2 d at 30° with shaking at 200 rpm. A total of 20 μl from each culture were then diluted using 80 μl of sterile water and spread onto YPD plates containing different doses of hydrogen peroxide. These plates were incubated at 30° for 4–6 d, so that slow-growing mutants would have enough time to form visible colonies. Glycerol freezer stocks were then made for mutants that formed visible colonies on doses at least 1 mM higher than the minimum inhibitory concentration (MIC) of their progenitor and stored at −80°. After this initial screen, all mutants were then phenotyped side-by-side with their respective progenitors across a broad range of hydrogen peroxide doses to confirm their increased tolerance. Mutants that grew on doses at least 0.5 mM higher than their progenitor were saved for downstream analysis. Fourteen BY × RM, nine RM × YPS, and 14 BY × YPS derived mutants met this requirement.

### Whole genome sequencing of progenitors and mutants

Archived mutants, as well as their progenitors, were inoculated into YPD liquid and outgrown for 2 d at 30°. For each mutant, DNA was extracted using the Qiagen DNeasy kit and a whole genome library was prepared using the Illumina Nextera kit. Each library was constructed using limited cycle PCR to add adapters to Nextera-treated genomic DNA according to manufacturer recommendations. Two unique 8-bp barcodes were incorporated into each library, so that dual indexed sequencing could be performed. Sequencing was conducted on an Illumina NextSeq 500 instrument at the USC Epigenome Center. Reads were then demultiplexed using custom Python scripts. The average per site coverages of the progenitors and mutants were 116× and 130×, respectively (see Note S1 in File S1).

### Analysis of sequencing data

To facilitate high confidence identification of mutations using the short-read sequencing data, progenitor-specific reference genomes were generated. Burrows–Wheeler Aligner (BWA-MEM) software (Li and Durbin 2009) was used to map reads from the progenitors to the BY genome. The parameters used for alignment were as follows: “bwa –mem –t 6 ref.fsa read1.f1 read2.fq > output.sam.” To remove duplicate reads, the rmdup command was implemented in SAMtools (Li *et al.* 2009). To create Mpileup files, SAMtools was then run with the command “samtools mpileup –f ref.fsa read.rmdp.srt.bam > output.mp.” We then identified genetic differences between a given progenitor and BY, integrated these differences into the BY genome, and then remapped reads for that progenitor to the modified genome sequence. This process was repeated for up to 10 cycles, and the output genome was then used as the reference for mapping reads from mutants derived from that progenitor. Reads from mutant strains were aligned to the progenitor reference genomes using BWA-MEM and the same parameters described above, followed by the generation of Mpileup files with SAMtools and the same parameters reported above. Point mutations and small indels were identified as differences from the corresponding progenitor that were seen in at least 90% of the reads at a particular site, whereas aneuploidies were detected based on changes in coverage across all or part of a chromosome. Custom Python scripts were used to identify these mutations, as well as to calculate per site or per genomic window sequencing coverage.

### Gene ontology enrichment analysis

Gene ontology (GO) analysis was carried out on the *Saccharomyces* Genome Database website (http://www.yeastgenome.org/cgi-bin/GO/goTermFinder.pl), using GO Term Finder version 0.83 with the molecular function category selected. All genes listed in Table S1 and Table S2 in File S1 were included in the analysis.

### PCR-mediated chromosomal deletion

Similar to Sugiyama *et al.* (2008), PCR-mediated chromosomal deletion (PCD) was implemented using constructs with three segments, in the following order: 300–600 bp of sequence homologous to the desired integration site on chromosome IV, a *kanMX* cassette, and a synthetic telomere seed sequence consisting of six repeats of the motif 5′-CCCCAA-3′ (Figure S2 in File S1). To generate this construct, the region containing the integration site was PCR amplified from genomic DNA using a reverse primer that was tailed with 30 bases of sequence identity to the *kanMX* construct. At the same time, *kanMX* was PCR amplified from a plasmid using a forward primer with 30 bases of sequence identity to the integration site and a reverse primer containing the synthetic telomere seed sequence. Integration site and *kanMX*/synthetic telomere seed sequences were then joined using overlap fusion PCR (Sugiyama *et al.* 2005). This was done by mixing the two products in equimolar fractions in combination with the forward primer for the integration site and the reverse primer for the *kanMX*/synthetic telomere seed sequence, and conducting PCR. The cycling parameters for overlap fusion PCR were as follows: initial denaturation at 98° for 3 min, followed by 30 cycles of 98° for 30 sec, 63° for 30 sec, and 72° for 1.5 min. The final step was a 5 min extension at 72°. Throughout this process, all PCR steps were implemented using NEB High-Fidelity Phusion polymerase and all PCR products were purified using either Qiagen QIAquick Gel Extraction or QIAquick PCR Purification kits. A standard lithium acetate–based technique was used to transform PCD constructs into cells, with ∼5 μg of construct employed (Gietz and Schiestl 2007). Transformants were recovered using selection for G418 resistance on YPD plates, verified by colony PCR or genome sequencing, and archived at −80° in glycerol solution. Primers used to construct PCD products are listed in Table S3 in File S1.

### Deletion of individual genes

Targeted gene deletions were performed using the *kanMX* cassette. Constructs for deleting *ADA2*, *ECM11*, *GUK1*, *NHX1*, and *UTP6* were generated by amplifying the *kanMX* cassette from a plasmid using tailed primers. Each tail contained 30–60 bases of sequence homology to the target gene’s flanking regions. Specifically, the forward and reverse primers were designed to have tails identical to the region immediately upstream of the translation start site and downstream of the stop codon, respectively. Regarding deletions of *PPN1*, *TOM1*, *TSA2*, *RPS17B*, *RPS18A*, *YDR455C*, and *YHP1* transformations using knockout cassettes tailed with only 60–120 bp of total homology to the targeted region were relatively inefficient. To increase the efficiency of these knockouts, gene deletion products were generated in a manner analogous to making the PCD constructs described above. Then, 300–600 bp of sequence targeting a region just upstream of the translation start site was fused to the *kanMX* cassette, with the caveat that the reverse primer for *kanMX* amplification was tailed with 30–60 bases of homology to a region just downstream of the gene being deleted. Additionally, several 100 bp of sequence up- and downstream of *TSA2* were deleted by the same method, using a modified targeting sequence and downstream homology tail. The same lithium acetate–based transformation methods described for PCD were used for all individual gene knockouts. Transformants were verified by colony PCR. Primers used for individual gene knockouts are listed in Table S4 in File S1.

### Plasmid-based increase of TSA2 copy number

Low-copy plasmids were constructed by cloning the complete *TSA2* locus (promoter, coding region, and 3′UTR) from the BY × RM haploid progenitor into the multiple cloning site of pRS410, a gift from Fred Cross purchased through Addgene (Addgene plasmid #11258). This plasmid contains *CEN6*/*ARS4*, which results in maintenance at low-copy number in *S. cerevisiae*, as well as *AmpR* and *KanR2*, which enable selection of transformed bacteria on ampicillin and transformed yeast on G418, respectively. Restriction sites for *Not*I and *Xho*I were added as 5′ tails to the primers used to amplify the *TSA2* locus. The pRS410 plasmid and modified *TSA2* PCR product were separately and sequentially digested with each enzyme at 37°, after which, the reactions were quenched by a 20-min incubation at 65°. After the second digestion step, the doubly digested plasmid and insert were ligated overnight. A small volume of the ligation reaction was then used to transform competent *Escherichia coli* obtained from Invitrogen. Transformants were selected on LB plates supplemented with ampicillin. Successful integration of the *TSA2* locus was confirmed by PCR amplifying DNA extracted from transformants using M13 primers, which flank the multiple cloning site. Plasmids were purified from transformed strains using the Qiagen midi plasmid purification kit and transformed into euploid progenitor strains. Based on Sanger sequencing, the cloned *TSA2* locus was found to have the same sequence as the BY × RM progenitor. Primers used for construction of plasmids are listed in Table S4 in File S1.

### qPCR analysis of TSA2 copy number

*TSA2* copy number was checked in overexpression transformants, as well as the BY × RM F_2_ progenitor and a BY × RM mutant disomic for chromosome IV, using qPCR. DNA was extracted from the transformants using the Qiagen QIAamp kit. Transformants were grown on selection plates containing G418. After 3 d at 30°, three colonies from each overexpression transformant and four colonies from the BY × RM progenitor and disomic mutant strains were transferred to 96-deep-well plates, with 800 μl of YPD, and incubated at 30° for 2 d with shaking, after which strains were either pinned onto YPD plates supplemented with hydrogen peroxide or transferred to 96-deep-well plates containing YPD broth supplemented with hydrogen peroxide. The remaining cells from each well were transferred to Eppendorf tubes, spun down at >13,000 rpm for 10 sec, after which the supernatant was removed and cells were resuspended in spheroplasting solution containing zymolyase. An RNase treatment was then performed to eliminate any transcripts that could potentially be amplified along with the genomic copies of *TSA2* and our reference gene *ACT1*. The KAPA SYBR Fast qPCR kit was used for qPCR analysis on the Agilent Mx3005p. 96-well semiskirted PCR plates were employed and Bio-Rad Microseal “C” Film was used to cover them. Cycling parameters were as follows: 95° for 2 min, followed by 40 cycles of 95° for 3 sec and 60° for 30 sec. At the end of each cycle, fluorescence measurements were then taken for each well. After the final cycle, a 10-min extension step was run at 72°, followed by a melting curve analysis from 55 to 95°, by 0.4° increments. After qPCR, products were checked on 1.5% agarose gels to ensure primer specificity. *TSA2* abundance in each sample was measured relative to *ACT1*. All experiments were conducted in at least biological triplicate. Primers used for qPCR are listed in Table S4 in File S1.

### Phenotyping of transformants

Transformants were outgrown for 2 d in 800 μl of YPD broth incubated at 30° with shaking. As controls for batch effects, each time one or more transformants were phenotyped, their euploid and aneuploid progenitors were also examined. After the liquid outgrowth step, strains were pinned onto YPD plates supplemented with different hydrogen peroxide doses. Alternatively, in some experiments, strains were then transferred to liquid media supplemented with a range of doses of hydrogen peroxide for 3 d, after which the OD630 of 50 μl of culture from each well diluted in 150 μl of water was measured using a plate reader. These strains were incubated at 30° with shaking. Each experiment was done in at least biological triplicate. Agar plates were incubated for 3 d at 30° and then imaged on a GelDoc imaging device using a 0.5 sec exposure time. MIC was determined as the lowest hydrogen peroxide dose at which a given strain could not grow.

### Data availability

All sequence data are available through the Sequence Read Archive under Bioproject number PRJNA338809, study accession number SRP081591, and sample accession numbers SRX2018688–SRX2018727 and SRX2037407–SRX2037410.

## Results and Discussion

### Screen for spontaneous mutations that increase hydrogen peroxide tolerance

A total of 24 independent cultures of each of the three haploid progenitor strains were examined after 2 d of outgrowth using selection on agar plates supplemented with hydrogen peroxide (see *Materials and Methods*). All mutants (37 total) that exhibited an increase in MIC at least 0.5 mM higher than their corresponding progenitor were analyzed further (see Figure S3, A–C in File S1; *Materials and Methods*). BY × RM, RM × YPS, and BY × YPS derived mutants were, on average, 2.3 mM (32%), 2.1 mM (25%), and 0.6 mM (7%) more tolerant than their progenitor, respectively (see Figure S3, A–C in File S1).

### The most frequently identified mutation is a chromosomal duplication

**Figure 1:**
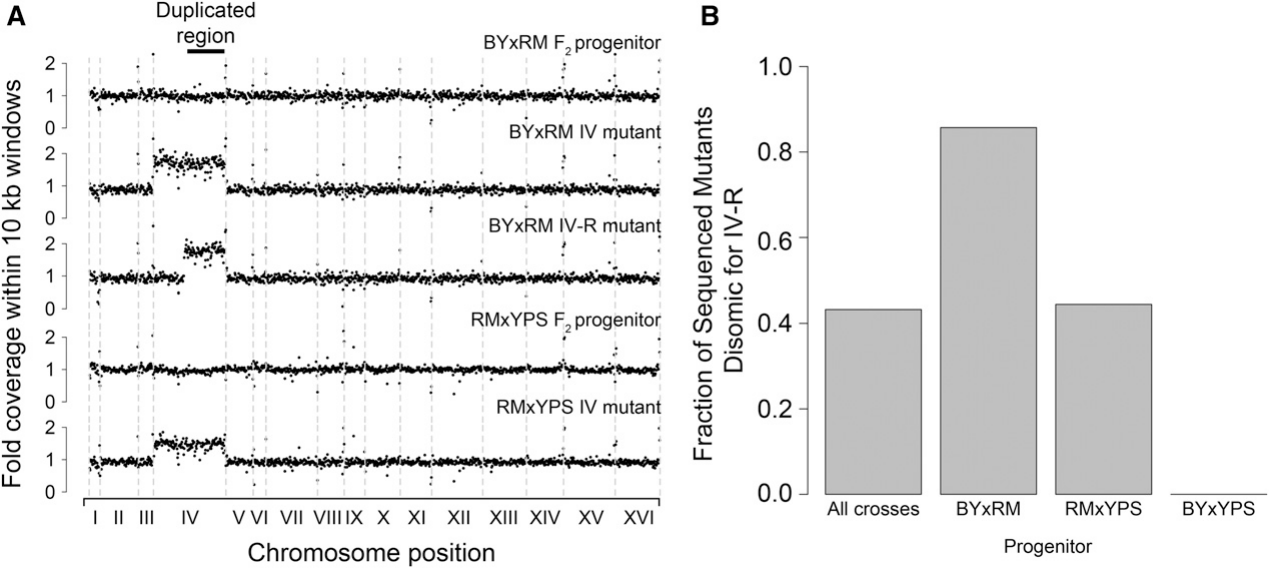
Chromosome IV duplication is the most frequent mutational event in a screen for spontaneous hydrogen peroxide resistance mutations. (A) Genome-wide coverage plots are provided for the BY × RM and RM × YPS F_2_ progenitors, as well as representative aneuploid or segmental duplication mutants derived from them. Site-specific coverages are scaled to the average per site coverage seen across the entire genome for a given strain. (B) Bar plots show the fraction of sequenced mutants that were disomic for the right arm of chromosome IV both across the entire screen and by individual progenitor.

Analysis of genome-wide sequencing coverage indicated that 43% of the mutants carried two complete copies of chromosome IV (Figure 1). No other aneuploidies were detected. The disomy was common among the BY × RM (79%) and RM × YPS derived (45%) mutants, but was absent from the BY × YPS derived mutants (Figure 1B). Given that the BY × RM and RM × YPS derived mutants also showed higher average gains in tolerance, this finding is consistent with duplication of chromosome IV conferring a sizable increase in tolerance (see Figure S3, A–C in File S1).

A single segmental duplication was also detected; this was found in a BY × RM derived mutant that possessed two copies of only the right arm of chromosome IV (chromosome IV-R; Figure 1A). The segmental duplication spanned ∼890 kb (58% of chromosome IV). Including this mutant, 86% of the BY × RM derived mutants were disomic for chromosome IV-R.

Additionally, 39 unique point mutations were identified among the mutants (see Table S1 and Table S2 in File S1). Twenty-two of these point mutations were nonsynonymous, eight were synonymous, and nine occurred in intergenic regions. Some point mutations were located in genes that affect hydrogen peroxide tolerance, including a SUMO E3 ligase involved in DNA repair (*MMS21*), an activator of cytochrome oxidase 1 translation (*PET309*), a subunit of cytochrome c oxidase (*COX1*), and a negative regulator of Ras-cAMP-PKA signaling (*GPB2*; see Table S1 and Table S2 in File S1). At false discovery rates of 0.17 or lower, no specific GO enrichments were seen among the genes harboring point mutations (see *Materials and Methods*). These results are consistent with our past finding that genetic perturbation of many different cellular processes can influence hydrogen peroxide tolerance (Linder *et al.* 2016), but could also have been driven by passenger mutations that do not play causal roles in hydrogen peroxide tolerance.

Duplication of chromosome IV-R was the most frequently detected mutation in our screen. However, strains carrying point mutations were found among the BY × RM and RM × YPS derived mutants that were as tolerant to hydrogen peroxide as strains containing the chromosome IV-R duplication (see Figure S3, A and B in File S1). In these genetic backgrounds, duplication of chromosome IV-R may occur more frequently than spontaneous point mutations that increase hydrogen peroxide tolerance (Kaya *et al.* 2015; Liu *et al.* 2015).

### Chromosome IV-R duplication is conditionally beneficial

**Figure 2:**
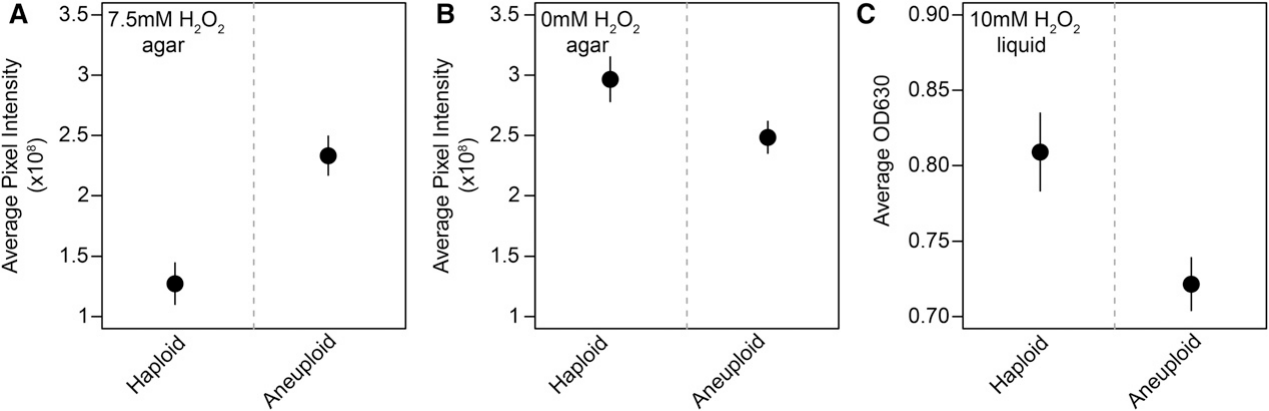
Duplication of chromosome IV is conditionally beneficial. (A) Replicates of a strain fully disomic for chromosome IV show significantly improved growth compared to euploid replicates when grown on agar plates supplemented with 7.5 mM hydrogen peroxide. However, when these strains are grown on agar plates with rich medium (B), euploid individuals show superior overall growth as compared to individuals disomic for chromosome IV. When both strains are exposed to 10 mM of hydrogen peroxide in liquid culture (C), euploid individuals also show higher growth than individuals disomic for chromosome IV. In (A) and (B), end-point colony pixel intensity measurements were generated using plate growth assays and image analysis in ImageJ. Mean and 95% confidence intervals are plotted. These measurements are based on four biological replicates per strain, which were each grown in three technical replicates (see Note S5 in File S1). In (C), individuals were exposed to hydrogen peroxide in liquid media for 3 d, after which the OD630 was measured for each culture. Measurements were again based on four biological and three technical replicates per strain (see *Materials and Methods*).

Chromosome IV-R duplication only conferred a growth benefit when hydrogen peroxide exposure occurred on agar plates (Figure 2A and Figure S4, A and B in File S1). In other conditions, chromosome IV-R duplication appeared to have a fitness cost. Similar to past reports that chromosomal duplications are usually deleterious (Pavelka *et al.* 2010; Sunshine *et al.* 2016), chromosome IV-R duplication reduced growth both on agar plates without hydrogen peroxide and in liquid medium containing hydrogen peroxide (Figure 2, B and C and Figure S4, A and B in File S1).

### Identification of a single region responsible for most of the effect of chromosome IV-R duplication

To map the conditional growth benefit of chromosome IV-R duplication to specific genes, we adapted a technique known as PCD, which involves eliminating segments of a chromosome that are distal to a centromere by inserting a drug resistance cassette linked to a synthetic telomere seed sequence (Figure S2 in File S1; *Materials and Methods*) (Sugiyama *et al.* 2005, 2008; Kaboli *et al.* 2016). Colony PCR was used both to confirm correct placement of the deletion cassette as well as to verify that a single copy of the deleted region remained (see *Materials and Methods*).

**Figure 3:**
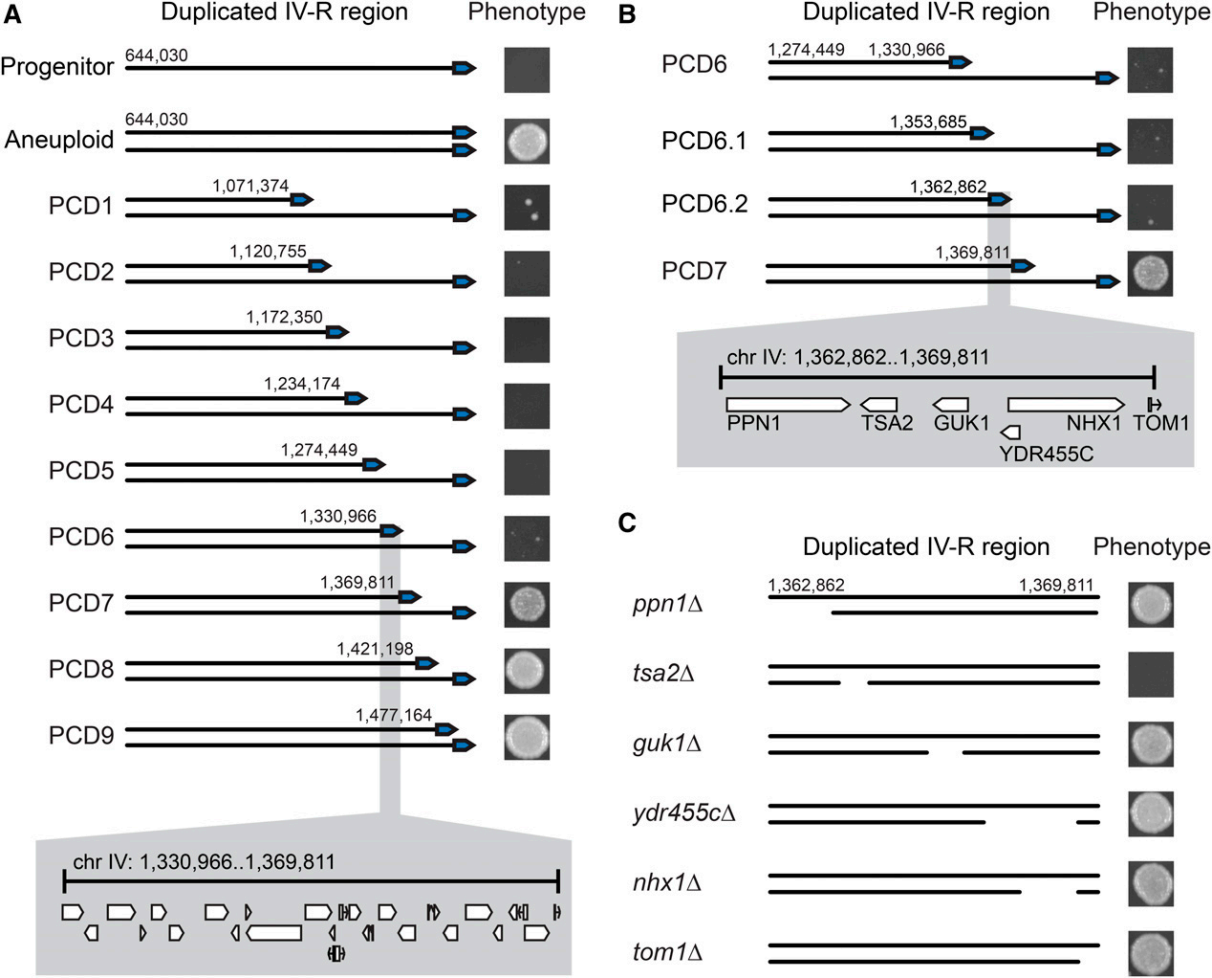
Genetic dissection of the chromosome IV duplication’s effect on hydrogen peroxide tolerance. (A) PCDs were staggered nearly every 50 kb along chromosome IV-R in a BY × RM derived aneuploid. Phenotyping of partially aneuploid strains generated by PCD identified a single genomic interval with a large effect on hydrogen peroxide tolerance when strains were examined at a dose of 7.5 mM. This ∼40 kb region contains 23 genes and three dubious ORFs. (B) Two additional PCD strains were generated within the previously mentioned window and examined at 7.5 mM. This narrowed the interval to 7 kb that contained five genes and a dubious ORF. (C) Individual gene deletions revealed that *TSA2* is largely responsible for the increase in tolerance conferred by duplication of chromosome IV-R. In (A and B), representative images of colonies grown at 7.5 mM of hydrogen peroxide are shown next to their associated PCD strains; numbers adjacent to the telomere indicate the starting position of each PCD on chromosome IV. In (C), representative images of colonies grown at 7.5 mM of hydrogen peroxide are again shown next to their associated individual gene deletion strains, with deleted regions indicated by gaps on the duplicated chromosome.

We first used PCD to delete the right half of chromosome IV-R from a BY × RM derived aneuploid (Figure 3A; *Materials and Methods*). This genetic change, which was confirmed by whole genome sequencing (see Figure S5 in File S1; *Materials and Methods*), caused a reversion to the hydrogen peroxide tolerance exhibited by the euploid BY × RM progenitor prior to the screen (see Figure S6A in File S1). This result indicates that the deleted chromosomal segment is required for the aneuploidy’s effect.

We next generated a panel of PCD strains, with large-scale deletions staggered, on average, every 50.7 kb along the distal half of chromosome IV-R. This led to the identification of a single 40 kb region that recapitulates most of the effect of the chromosome IV-R disomy (Figure 3A and Figure S6A in File S1). It is important to note that, although chromosome-scale deletions of this 40 kb region appeared to phenocopy the progenitor at certain hydrogen peroxide doses (Figure 3, A and B), the average MICs of these strains were higher than that of their progenitor (Figure S6, A and B in File S1). Possible explanations for this result include the presence of one or more additional point mutations that contribute to hydrogen peroxide tolerance in the mutant, an additional gene or regulatory element on chromosome IV-R whose dosage contributes to tolerance, and nonlinear relationships between DNA content and hydrogen peroxide tolerance, as the chromosome-scale deletion strains remain aneuploid at a large portion of chromosome IV (see Note S2 in File S1). We note that batch effects resulted in slight differences in the growth of the euploid progenitor at 7.5 mM hydrogen peroxide across separate experiments, but that growth differences between genotypes remained qualitatively consistent (Figure 3A, Figure S4A in File S1, and Figure S10 in File S1).

### Duplication of TSA2 mediates the conditionally beneficial effect of the aneuploidy

To further resolve this window, we performed two additional PCD transformations, which fine-mapped the causal interval to roughly 7 kb, spanning positions 1,362,862 to 1,369,812 bp (Figure 3B and Figure S6B in File S1). This interval contains five genes—the polyphosphatase *PPN1*, the cytoplasmic thioredoxin peroxidase *TSA2*, the guanylate kinase *GUK1*, the ion exchanger *NHX1*, and the E3 ubiquitin ligase *TOM1*—as well as a dubious ORF (*YDR455C*). We used standard techniques to individually delete each of these genes from a BY × RM derived aneuploid, again using colony PCR to verify both correct placement of the deletion cassette and that a single copy of the gene remained (see *Materials and Methods*). Also, because telomeres can influence the transcription of genes >20 kb away (Gottschling *et al.* 1990; Aparicio and Gottschling 1994), we deleted the six genes upstream of *PPN1* (Figure 3B and Figure S7 in File S1).

The only gene deletion that showed a phenotypic effect was *TSA2*, which encodes a cytoplasmic thioredoxin peroxidase (Figure 3, B and C and Figure S7 in File S1) (Gasch *et al.* 2000; Park *et al.* 2000; Wong *et al.* 2002; Munhoz and Netto 2004; Ogusucu *et al.* 2007; Nielsen *et al.* 2016). Previous work has shown that deleting *TSA2* leads to a decrease in hydrogen peroxide tolerance (Wong *et al.* 2002). Loss of the *TSA2* coding region eliminated the majority of the aneuploidy’s effect (Figure 3C and Figure S7 in File S1), proving a causal role for *TSA2* in the conditional benefit conferred by chromosome IV-R duplication. Knockout of *TSA2* in other mutants, including a fully disomic BY × RM mutant, a partially disomic BY × RM mutant, and a fully disomic RM × YPS mutant, confirmed that the effect of *TSA2* was reproducible across different aneuploid individuals recovered from our screen (Figure S8 in File S1; see Note S3 in File S1).

### Plasmid-based overexpression of TSA2 increases tolerance of hydrogen peroxide

**Figure 4:**
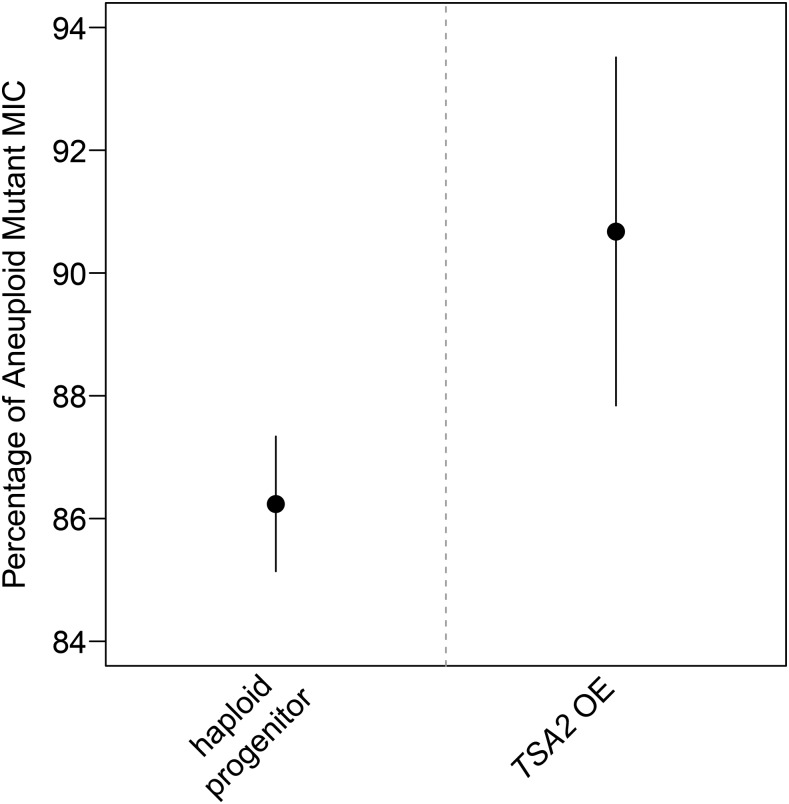
Plasmid-based overexpression of *TSA2* partially recapitulates the effect of having two chromosomal copies of this gene. The entirety of the *TSA2* locus was cloned into a *CEN* plasmid that was then transformed into the haploid progenitor (see *Materials and Methods*). Two independently generated *TSA2* overexpressing strains were screened for hydrogen peroxide tolerance alongside the haploid progenitor and disomic strain. Shown are 95% confidence intervals for the MIC of the overexpressing strains as a percentage of the MIC of the aneuploid strain. These measurements are based on three biological replicates, which were each grown in three technical replicates.

To further confirm that an increase in copy number of *TSA2* has a beneficial effect on hydrogen peroxide tolerance, the complete *TSA2* locus was cloned into a low-copy *CEN* plasmid, which was subsequently transformed into the BY × RM euploid progenitor (see *Materials and Methods*). Increased copy number of *TSA2* in the euploid progenitor was confirmed through qPCR (Figure S9 in File S1; *Materials and Methods*). Plasmid-based overexpression of *TSA2* in the euploid progenitor resulted in a significant increase in tolerance (Figure 4 and Figure S10 in File S1). However, the low-copy number plasmid did not fully recapitulate the effect of having a second chromosomal copy of the gene, further suggesting that *TSA2* does not fully explain the effects of chromosome IV disomy on hydrogen peroxide tolerance (Figure 4, Figure S10 in File S1, and Figure S11 in File S1). In line with this possibility, *TSA2* overexpression strains did not show the increased susceptibility to hydrogen peroxide that was exhibited by aneuploid mutants in liquid media (Figure S12 in File S1).

### Conclusion

Previously, we found that the maximal hydrogen peroxide tolerances of segregants from the BY × RM, BY × YPS, and RM × YPS crosses are comparable (see Figure S1 in File S1) (Linder *et al.* 2016), suggesting that the level of resistance achievable through natural genetic variation may be constrained to some degree. To surpass the levels of tolerance seen in our prior study, we conducted a screen for spontaneous mutations that confer higher hydrogen peroxide resistance than we previously observed. We employed segregants that had maximal tolerances as the progenitors in our mutagenesis screen because these individuals carry combinations of natural genetic variants that lead to resistance and we wanted to find mutations that provide even greater tolerance than these allele combinations.

Duplication of chromosome IV-R was the most common mutation in our screen. As with other chromosomal duplications (Pavelka *et al.* 2010; Sunshine *et al.* 2015), the beneficial effect of the chromosome IV-R disomy is conditional: it depends on both the presence of hydrogen peroxide and exposure to hydrogen peroxide on agar plates, and may be dependent on genetic background. Regarding this latter point, the present data cannot differentiate whether the progenitors in our screen varied in their genome stabilities or in their abilities to beneficially utilize the chromosome IV-R disomy. Assessment of genotype at *TSA2* indicates that preexisting variation at this locus probably does not explain why we observed chromosome IV aneuploids in the BY × RM and RM × YPS crosses, but not the BY × YPS cross (see Note S4 in File S1).

Using chromosome- and gene-scale deletions, we determined that increased copy number of a single detoxifying gene, *TSA2*, explains the majority of the benefit conferred by duplication of chromosome IV-R. *TSA2* is unique among budding yeast’s cytoplasmic thioredoxin peroxidases, as it is the only one that shows markedly increased activation in response to hydrogen peroxide (Gasch *et al.* 2000; Park *et al.* 2000; Wong *et al.* 2002; Munhoz and Netto 2004; Ogusucu *et al.* 2007; Nielsen *et al.* 2016). Our results are consistent with recent studies from other groups showing that typically, a small number of genes, usually one or two, mediate the conditionally beneficial effects of aneuploidies (Pavelka *et al.* 2010; Chen *et al.* 2012a; Kaya *et al.* 2015; Liu *et al.* 2015).

In summary, our work speaks to challenges in enhancing particular traits using natural genetic variation, spontaneous mutations, or a combination of the two. Indeed, the maximal trait values achievable through natural genetic variation may be limited because of both the specific alleles present in a population and features of a system that prevent extreme phenotypic levels from occurring. Trying to overcome these limits may be achievable using spontaneous mutations, which have not experienced the same selection pressures as natural variants and may be more likely to have large effects. However, our work suggests that the most likely mutational event to underlie such phenotypic increases is chromosomal duplication. Although these duplications have a limitation in that they can be easily lost (Berman 2016), they may represent a transient state that can facilitate the acquisition of other mutations that provide a more permanent solution to stressful conditions (Yona *et al.* 2012).

## Supplementary Material

Supplemental material is available online at www.g3journal.org/lookup/suppl/doi:10.1534/g3.117.300069/-/DC1.
