# Supplementary material for: The Stress-Inducible Peroxidase TSA2 Underlies a Conditionally Beneficial Chromosomal Duplication in Saccharomyces cerevisiae
Source: G3 (Bethesda). 2017 Jul 26;7(9):3177–84. doi: 10.1534/g3.117.300069 (PMC5592942; doi:10.1534/g3.117.300069)
Supplement: Supplementary file 1 [file 3177FigureS1.docx]

**Supplementary Information**


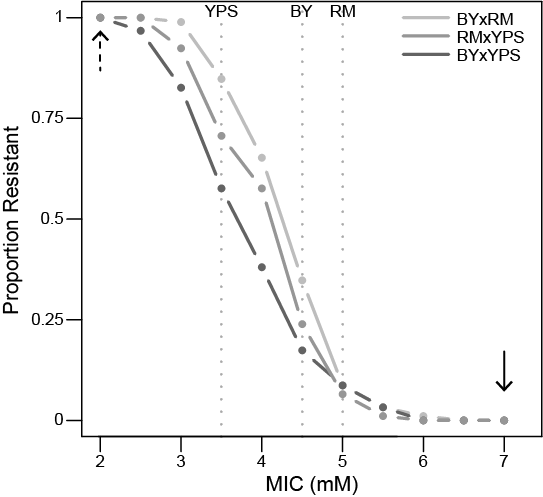


**Figure S1.** In our previous study, progeny generated from all pairwise crosses of BY, RM, and YPS reveal that hydrogen peroxide tolerance is strictly bounded in natural populations. Tolerances of the parental strains are depicted as vertical dashed lines. The minimal trait value for F_2_ progeny from each cross was approximately 2mM (highlighted by a dashed arrow), while the maximal trait value was approximately 7mM (highlighted by a solid arrow). Modified from {Linder et al, 2016}.


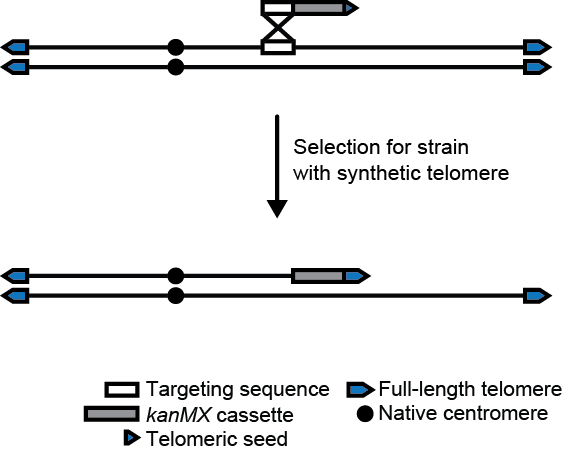


**Figure S2.** PCR-mediated chromosome deletion (PCD) was used to eliminate duplicated chromosomal segments from a BYxRM-derived aneuploid that possessed two complete copies of Chromosome IV. As described in the Methods, each PCD construct consisted of a sequence identical to a particular site on the chromosome, a *kanMX* marker providing resistance to G418, and a synthetic telomere seed sequence.


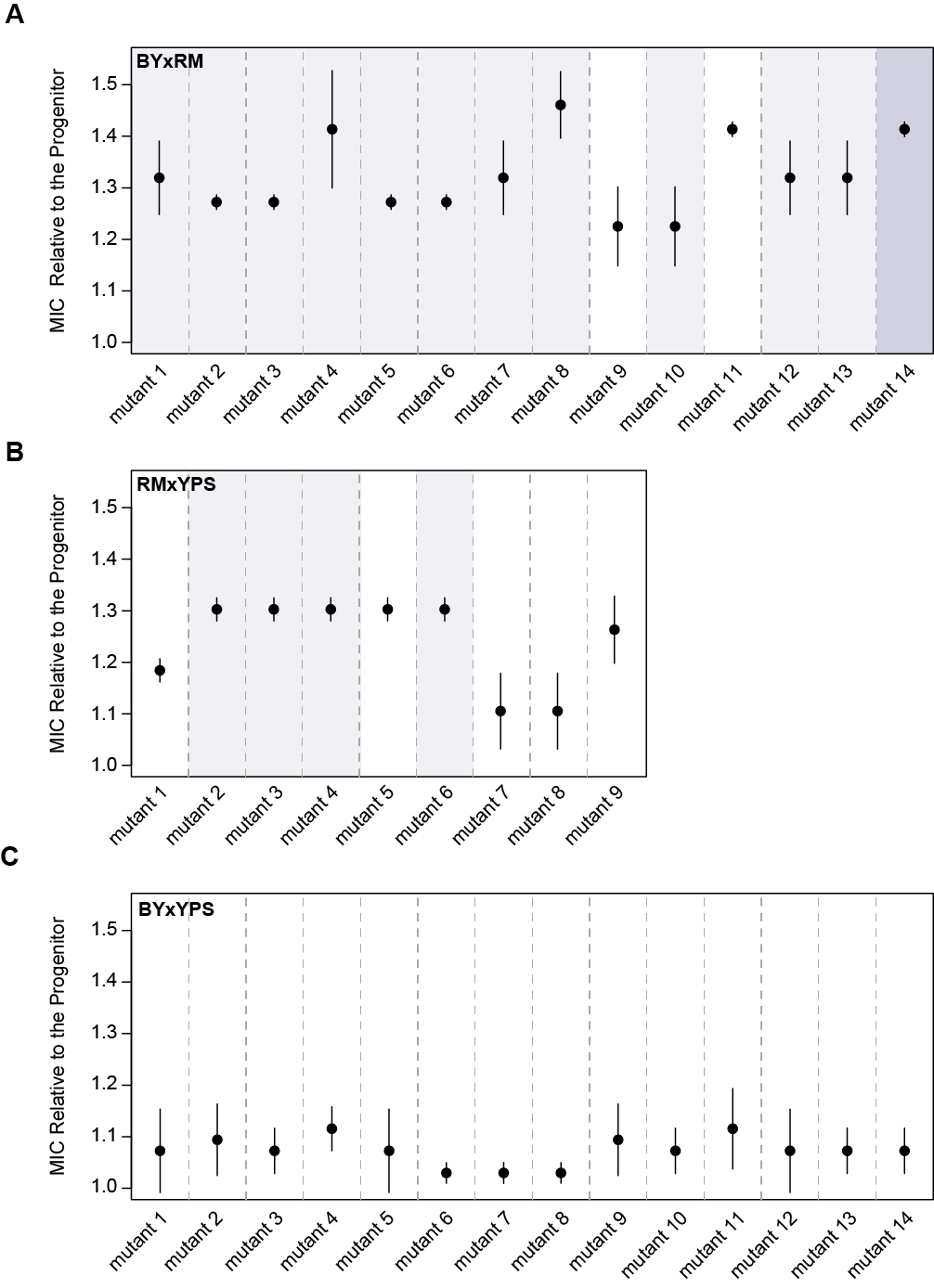


**Figure S3.** Screen to verify increased tolerance of mutants relative to their progenitors. Panels (**A**-**C**) depict phenotyping results of mutants generated from the BYxRM, RMxYPS, and BYxYPS crosses, respectively. Mutants highlighted with light gray boxes were found to be disomic for Chromosome IV, while the BYxRM mutant highlighted by a dark gray box was found to carry a segmental duplication of the distal part of Chromosome IV. Each mutant was phenotyped using three biological replicates. Shown are 95% confidence intervals for the MIC of each mutant as a fraction of the MIC of the corresponding progenitor strain.


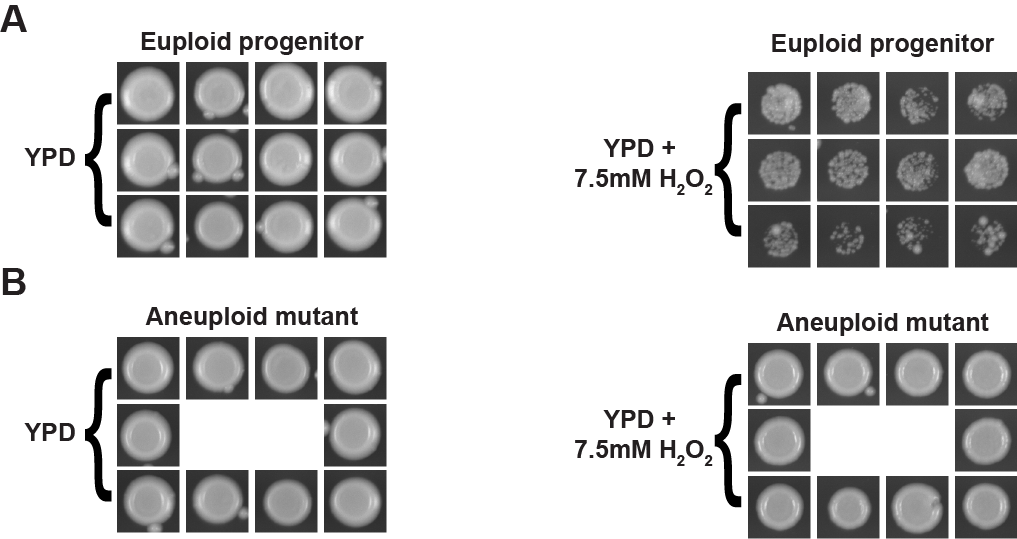


**Figure S4. (A)** and **(B)** depict the strains used to calculate pixel intensities in Figure 2A and B of the main text. All strains shown above were phenotyped in the same assay by pinning replicates onto agar plates with or without hydrogen peroxide supplementation. In **(A)** and **(B)**, four biological replicates were phenotyped. All biological replicates were assayed in three technical replicates. Two aneuploid mutant strains showed abnormal growth and were precluded from this analysis (see Note S5).

**
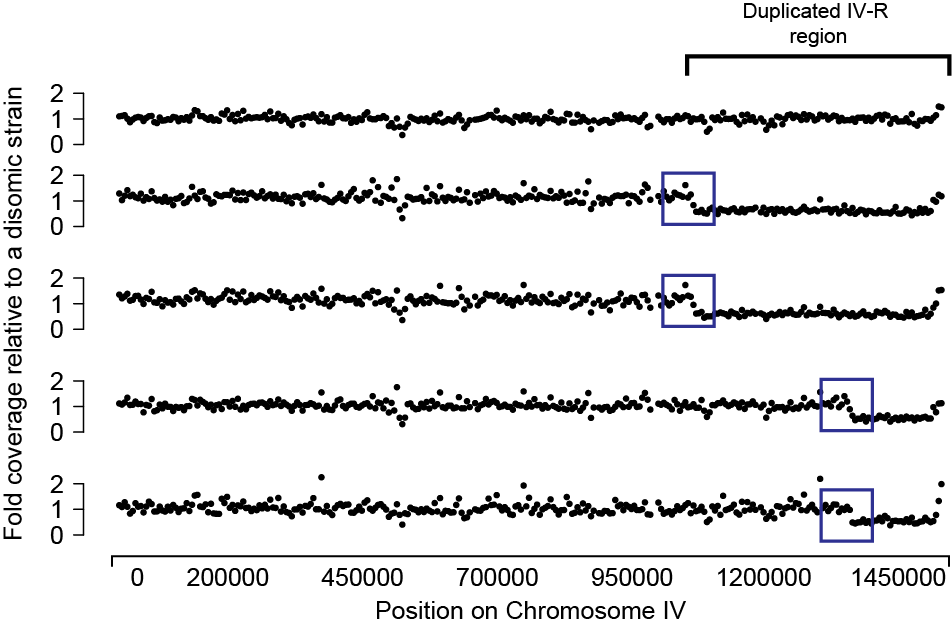
**

**Figure S5.** In order to empirically show that the chromosomal-scale deletions were removing a single copy of the targeted regions, four deletion strains were sequenced on an Illumina NextSeq500 (Methods). The top plot depicts a strain completely disomic for Chromosome IV as a comparison, while the two plots just below depict two independent PCD1 strains. The two bottom plots show two independent PCD6.2 strains. Blue boxes highlight the targeted region of Chromosome IV, with the remaining downstream sequence showing coverage half that of the remaining disomic part of the Chromosome.


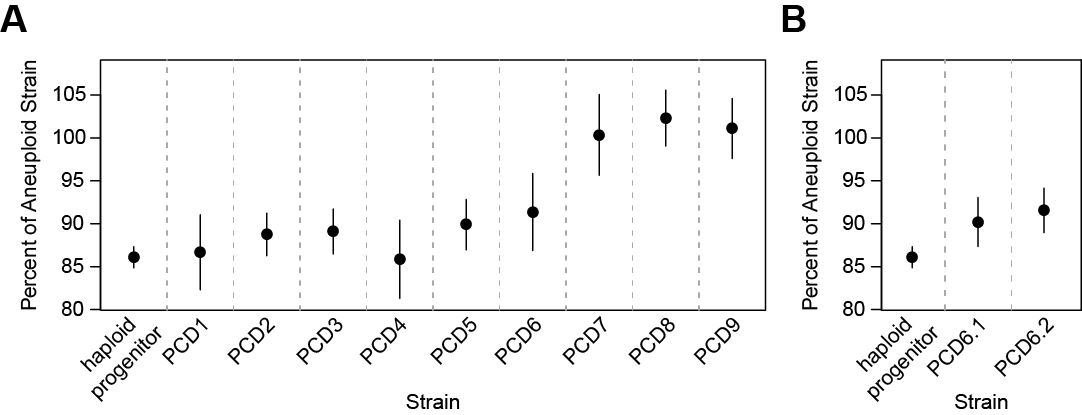


**Figure S6.** The hydrogen peroxide tolerances of all PCD strains are shown as a fraction of their aneuploid progenitors’ tolerance. Each panel represents a separate experiment in which biological replicates of the haploid progenitor as well as a fully disomic strain were plated along with the deletion strains to serve as controls. The number after PCD (PCR-mediated chromosomal deletion strains) represents the order in which these strains were used to map the causal locus. (**A**) PCD1-6 all led to a significant decrease in tolerance, while PCD7-9 did not show any significant loss in tolerance, implying that the genomic region between PCD6 and PCD7 has a large role in the aneuploidy’s effect. (**B**) PCD6.1 and PCD6.2 were constructed later to fine map the region. All plots show the mean and 95% confidence intervals for the MIC of each PCD strain as a percentage of the MIC of the aneuploid strains phenotyped in the same experiment. These measurements are based on at least 6 biological replicates per strain. Tolerance of the haploid progenitor relative to the fully disomic strain was generated as in Figure 4 of the main text.


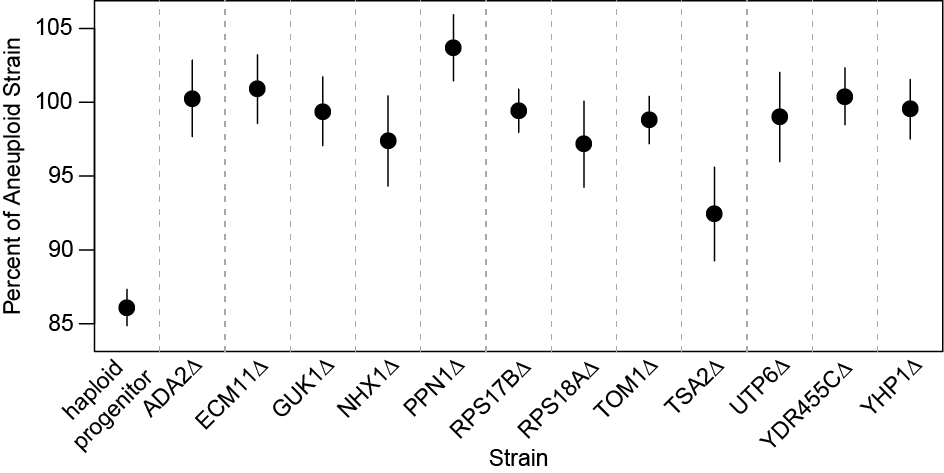


**Figure S7.** All individual gene deletions that were tested after fine-mapping the causal locus are shown. Each panel again represents an individual experiment with the haploid progenitor and disomic strain present as controls. Shown are 95% confidence intervals for the MIC of each individual deletion strain as a percentage of the MIC of the aneuploid strain. These measurements are based on at least 12 biological replicates. Tolerance of the haploid progenitor relative to the fully disomic strain was generated as above.


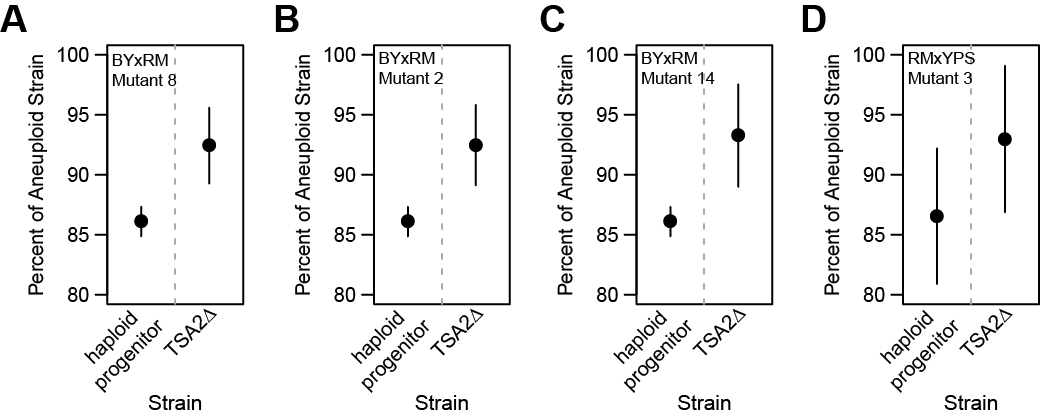


**Figure S8.** To verify that *TSA2* generally had an effect in mutants with a duplicate copy of IV-R, knock-outs of this gene were generated in multiple sequenced mutants. Each panel again represents an individual experiment with the haploid progenitor and disomic strain present as controls. The effect of deleting *TSA2* in the same aneuploid mutant as used in previous figures is reproduced in panel **(A)** to make comparisons between backgrounds easier. Panel **(B)** depicts the effect of deleting *TSA2* in a different fully disomic BYxRM mutant, while **(C)** depicts the effect of this deletion in the partially disomic BYxRM mutant. The effect of the deletion in an RMxYPS disomic mutant is shown in **(D)**. Shown are 95% confidence intervals for the MIC of each individual deletion strain as a percentage of the MIC of the aneuploid strain. These measurements are based on at least 3 biological replicates. Tolerance of the BYxRM haploid progenitor relative to the aneuploid BYxRM mutants was generated as above.


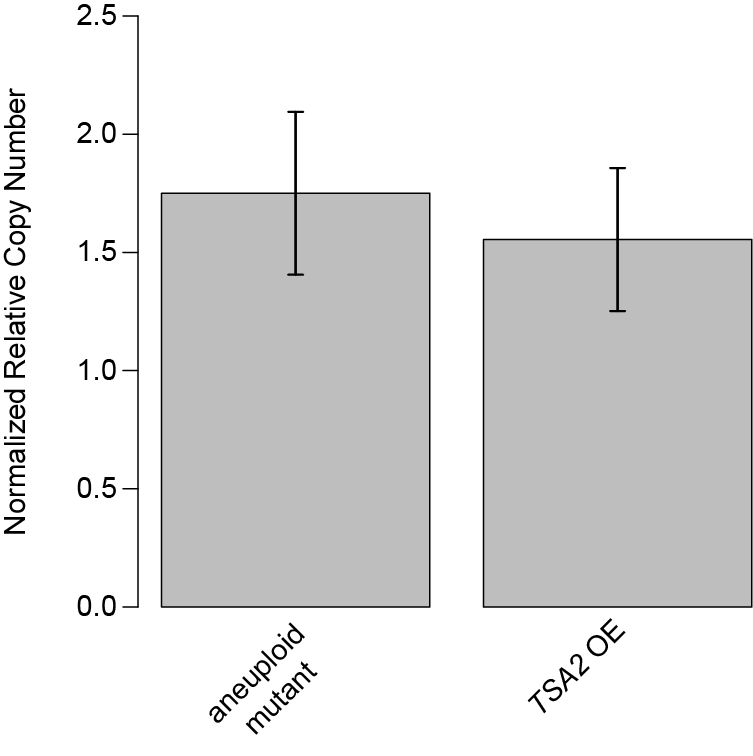


**Figure S9.** qPCR was used to confirm the increased copy number of *TSA2* in euploid strains transformed with a low-copy *CEN* plasmid containing the entire *TSA2* locus (Methods). Measurements of *TSA2* copy number in the over-expressing euploid progenitor are based on six biological replicates, each of which were queried as three technical replicates. For comparison, the euploid progenitor and a fully disomic mutant were assayed on the same plate in biological quadruplicate, each of which were queried as three technical replicates (Methods). The copy number of *TSA2* for all strains was normalized against *ACT1* (Methods). The above plot depicts the relative copy number of *TSA2* as compared to the euploid progenitor for both the fully disomic mutant and the *TSA2* overexpressing euploid strains. Both strains display a copy number that is significantly higher than that of the euploid progenitor.

**
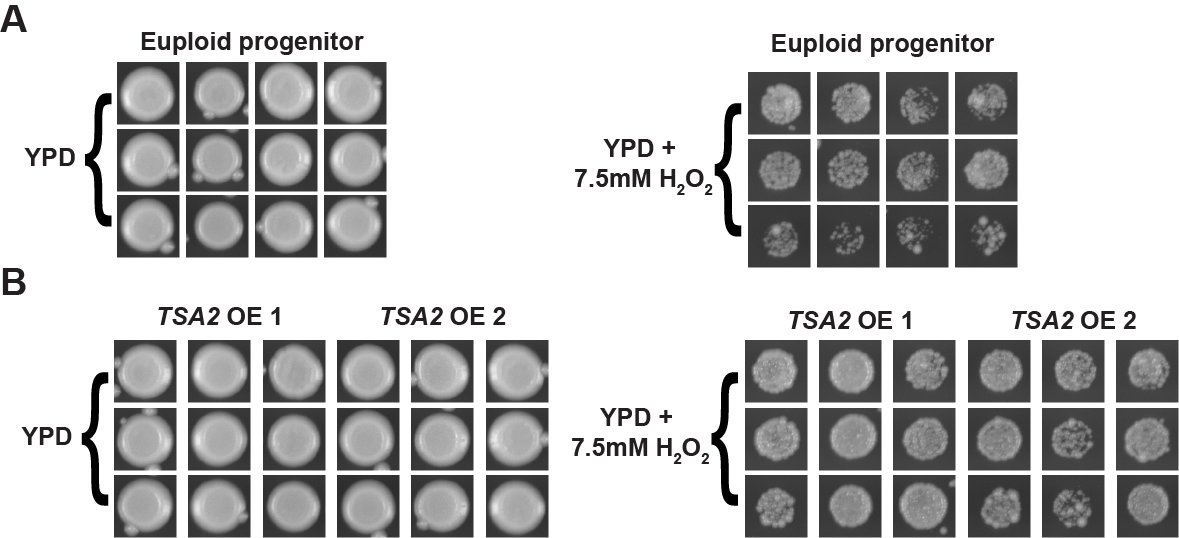
**

**Figure S10.** All strains shown above were phenotyped in the same assay by pinning replicates onto agar plates with or without hydrogen peroxide supplementation. In **(A)**, four biological replicates were phenotyped. In **(B)**, three biological replicates of two independently generated *TSA2* overexpressing strains are shown that were used to infer the MICs for Figure 4 of the main text. All biological replicates shown above were assayed in three technical replicates.

**
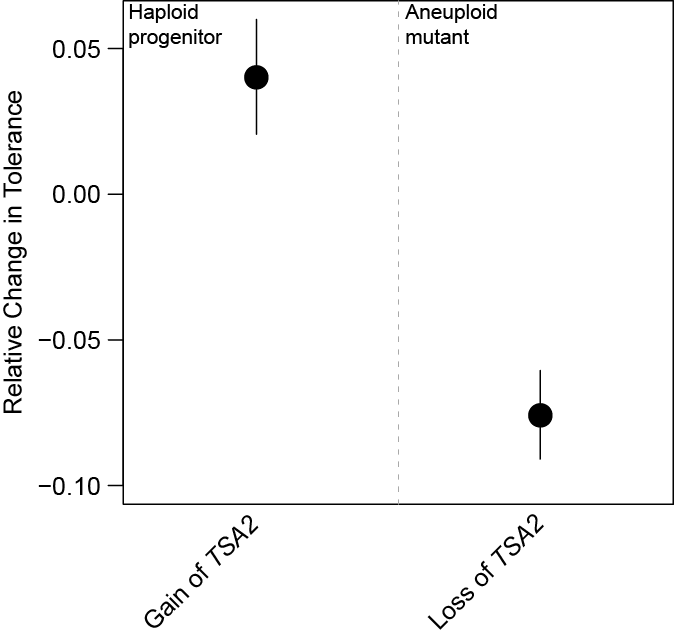
**

**Figure S11.** Gain of an extrachromosomal copy of *TSA2* leads to an increase in tolerance that is roughly half of the magnitude of the effect of losing the additional copy of *TSA2* present in IV mutants on agar plates. Each panel again represents an individual experiment with the haploid progenitor and disomic strain present as controls. The left panel was generated by determining the relative MIC of *TSA2* over-expressing strains as compared to the euploid progenitor, while the right panel was generated by determining the relative MIC of disomic strains with one copy of *TSA2* deleted as compared to strains fully disomic for Chromosome IV. Shown are 95% confidence intervals for the MIC.

**
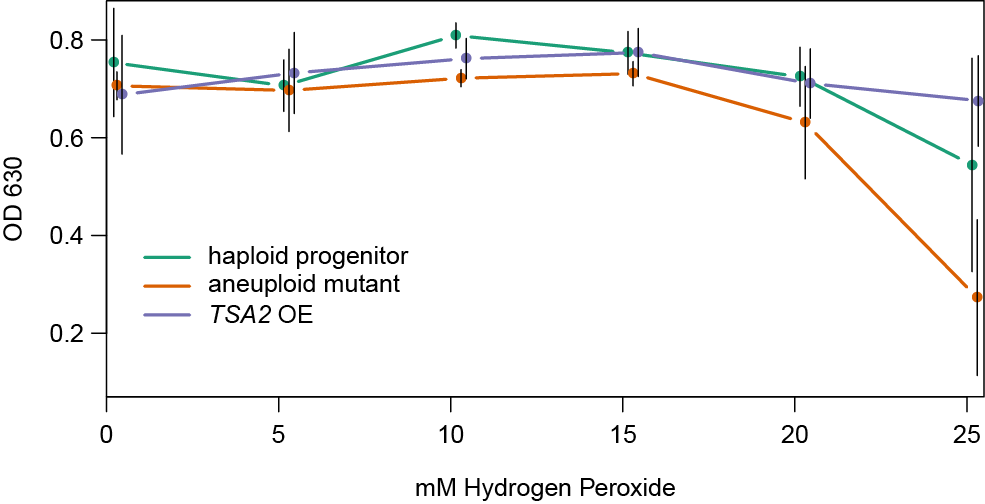
**

**Figure S12.** The haploid progenitor, a IV mutant, and two *TSA2* over-expressing strains were treated with a range of doses of hydrogen peroxide in liquid media for three days, after which OD630 measurements were taken (Methods). Shown are 95% confidence intervals for the OD630 of each strain. These measurements are based on at least 3 biological replicates, which were each grown in three technical replicates­.

**Table S1.** All non-structural mutations detected in mutants derived from the BYxRM, RMxYPS, and BYxYPS crosses.

| **Chr** | **Pos** | **Refpos*** | **Gene** | **Lesion**  ****** | **Strain**  ******* | **Location**  ******** | **Effect**  ********* |
| --- | --- | --- | --- | --- | --- | --- | --- |
| 1 | 40914 | 40916 | *GPB2* | G | BYxRM mutants 3, 8, and 12 | CDS | NS |
| 1 | 57765 | 57765 | *YAL044W-A* | T | BYxRM mutant 1 | CDS | NS |
| 2 | 144094 | 144084 | *URA7* | G | BYxYPS mutant 4 | CDS | NS |
| 2 | 483354 | 483244 | *GRS1* | T | BYxRM mutant 9 | CDS | NS |
| 2 | 593896 | 593740 | *SMP1* | A | BYxRM mutant 8 | CDS | S |
| 4 | 16532 | 16522 | *THI13* | T | BYxYPS mutant 5 | CDS | S |
| 4 | 305868 | 305840 | *SUB2* | T | BYxYPS mutant 9 | CDS | NS |
| 4 | 461128 | 460794 | *SOK1* | A | RMxYPS mutant 9 | CDS | NS |
| 4 | 822859 | 822773 | *SCC2* | T | BYxRM mutant 13 | CDS | NS |
| 5 | 38482 | 38484 | *CIN8* | C | BYxYPS mutant 8 | CDS | NS |
| 5 | 91037 | 90931 | *SPF1* | G | RMxYPS mutant 1 | CDS | NS |
| 5 | 118146 | 118032 | *RPR1* | A | RMxYPS mutant 1 | ncRNA_gene | U |
| 5 | 120972 | 120856 | *MMS21* | A | RMxYPS mutant 1 | CDS | NS |
| 6 | 83148 | 83128 | *STE2* | G | RMxYPS mutant 1 | CDS | NS |
| 7 | 93906 | 93900 | *YPT32* | T | BYxYPS mutant 4 | CDS | NS |
| 7 | 354692 | 354636 | *SCY1* | T | BYxYPS mutant 9 | CDS | NS |
| 7 | 857992 | 857570 | *TIM13* | T | RMxYPS mutant 2 | I | U |
| 8 | 528379 | 528255 | *FLO5* | G | BYxRM mutant 14 | CDS | NS |
| 9 | 348524 | 348522 | *BET1* | A | BYxYPS mutant 5 | I, promoter | U |
| 10 | 65759 | 65755 | *UBP12* | C | BYxRM mutant 1 | CDS | NS |
| 11 | 208307 | 208097 | *RRN3* | C | BYxRM mutants 1, 6, 11, and 13 | I, promoter | U |
| 11 | 521871 | 521501 | *SHB17* | A | BYxRM mutant 5 | CDS | S |
| 11 | 625954 | 625916 | *PCC1* | T | BYxYPS mutant 6 | Intronic | U |
| 12 | 185005 | 185003 | *SDO1* | C | BYxRM mutant 12 | I, promoter | U |
| 12 | 268070 | 267996 | *PET309* | T | RMxYPS mutant 5 | CDS | NS |
| 12 | 701887 | 701823 | *YLR278C* | A | BYxRM mutant 11 | CDS | NS |
| 13 | 141798 | 141788 | *SMA2* | T | BYxYPS mutant 8 | I, promoter | U |
| 13 | 531606 | 531434 | *POM152* | T | BYxRM mutant 12 | CDS | NS |
| 13 | 695503 | 695287 | *SCJ1* | A | RMxYPS mutant 9 | I, promoter | U |
| 14 | 40328 | 40298 | *HXT14* | G | BYxRM mutant 11 | CDS | NS |
| 14 | 153094 | 153064 | *POL2* | G | BYxRM mutant 13 | CDS | S |
| 14 | 467526 | 467492 | *MKT1* | A | BYxRM mutant 4 | CDS | NS |
| 15 | 214314 | 214288 | *MAM3* | A | BYxYPS mutant 2 | CDS | S |
| 15 | 391070 | 391020 | *HMS1* | T | BYxYPS mutant 4 | CDS | NS |
| 15 | 819835 | 819779 | *DSE3* | T | BYxYPS mutant 1 | CDS | NS |
| 16 | 400233 | 400227 | *MOT1* | A | BYxYPS mutant 8 | CDS | S |
| 16 | 776403 | 776315 | *YPRW delta14* | C | BYxRM mutants 1, 2, 3, 4, 6, 7, 8, 11, 12, 13 | Ty1 LTR | U |
| 16 | 835165 | 835077 | *PIN3* | C | BYxRM mutant 4 | CDS | S |
| 17 | 20568 | 20570 | *COX1* | A | BYxYPS mutant 6 | CDS | S |

***** This refers to the corresponding genomic position in the lab reference BY strain.

****** The specific base alteration that occurred

******* Strains in which the mutation was detected in. Strains that begin with ‘Br’ are derived from the BYxRM cross, strains that begin with ‘Ry’ are derived from the RMxYPS cross, while strains that begin with ‘By’ are derived from the BYxYPS cross.

******** The genomic context of each mutation. ‘CDS’, coding sequence of a gene; ‘I’, intergenic region**; ‘**promoter’, 5’-upstream region of a gene; ‘Intronic’, within an intron; ‘nc_RNA_gene’, non-coding RNA; ‘Ty1 LTR’, Ty1 retrotransposon.

********* The effect of each mutation. ‘NS’, non-synonymous change; ‘S’, synonymous change; ‘U’, unknown.

**Table S2.** All non-structural mutations detected in mutants derived from the BYxRM, RMxYPS, and BYxYPS crosses arranged by the strain these mutations were detected in.

| **Strain** | **Gene(s) Mutated** |
| --- | --- |
| BYxRM mutant 1 | *RRN3; UBP12; YAL044W-A;YPRW delta14* |
| BYxRM mutant 2 | *YPRW delta14* |
| BYxRM mutant 3 | *YPRW delta14; GPB2* |
| BYxRM mutant 4 | *MKT1; PIN3; YPRW delta14* |
| BYxRM mutant 5 | *SHB17* |
| BYxRM mutant 6 | *RRN3; YPRW delta14* |
| BYxRM mutant 7 | *YPRW delta14* |
| BYxRM mutant 8 | *GPB2; SMP1; YPRW delta14* |
| BYxRM mutant 9 | *GRS1* |
| BYxRM mutant 10 |  |
| BYxRM mutant 11 | *HXT14; RRN3; YLR278C; YPRW delta14* |
| BYxRM mutant 12 | *GPB2; POM152; SDO1; YPRW delta14* |
| BYxRM mutant 13 | *POL2; RRN3; SCC2; YPRW delta14* |
| BYxRM mutant 14 | *FLO5* |
| BYxYPS mutant 1 | *DSE3* |
| BYxYPS mutant 2 | *MAM3* |
| BYxYPS mutant 4 | *HMS1; URA7; YPT32* |
| BYxYPS mutant 5 | *BET1; THI13* |
| BYxYPS mutant 6 | *COX1; PCC1* |
| BYxYPS mutant 8 | *CIN8; MOT1; SMA2* |
| BYxYPS mutant 9 | *SCY1; SUB2* |
| RMxYPS mutant 1 | *SPF1; RPR1; MMS21; STE2* |
| RMxYPS mutant 2 | *TIM13* |
| RMxYPS mutant 5 | *PET309* |
| RMxYPS mutant 9 | *SCJ1; SOK1* |

**Table S3.** Primers used to make the PCD constructs and check for proper placement.

| **Primer** | **Sequence(5'-3')** |
| --- | --- |
| Kan_overlap-FP | **GGCCGCCAGCTGAAGCTTCGTACGCTGCAGCCTTGACAGTCTTGACGTGC *** |
| Kan_overlap-RP | **CCCCAACCCCAACCCCAACCCCAACCCCAACCCCAACGCACTTAACTTCGCATCTG **/***** |
| C4S1_del-FP **** | CCACGAATATCCATCGTTGTTT |
| C4S1_del-RP | **CTGCAGCGTACGAAGCTTCAGCTGGCGGCC**AGCCCACGATGAACTCTAAACT |
| C4S1_del_chk-FP ***** | GTGCAGCAGTTTCCTTGGAG |
| C4S1_del_HC-FP ****** | TTCCTCATGCTCGAAGGCTG |
| C4S1_del_HC-RP | GAGAATCGATGGCACCTTTCT |
| C4S2_del-FP | TCTACGCTTACCAGATATAGATGC |
| C4S2_del-RP | **CTGCAGCGTACGAAGCTTCAGCTGGCGGCC**AGAAGGTTAAGCTCGTAAATGGAGT |
| C4S2_del_chk-FP | CGAGGTCTGTTAAAATTGATATGCG |
| C4S3_del-FP | TGGAAAATGCTGGCAAAGTGT |
| C4S3_del-RP | **CTGCAGCGTACGAAGCTTCAGCTGGCGGCC**TCTCCTTGACCAATGCACGT |
| C4S3_del_chk-FP | ACTGTGGCATTTCTAAACGGG |
| C4S4_del-FP | AGCTCTATTTGTTAGCGTGGA |
| C4S4_del-RP | **CTGCAGCGTACGAAGCTTCAGCTGGCGGCCC**TTTGACGTCTTGGCTCCCA |
| C4S4_del_chk-FP | TCCATTGTCATCATCCCCGTC |
| C4S5_del-FP | CCGTGGCTTGTAATTTTCGGG |
| C4S5_del-RP | **CTGCAGCGTACGAAGCTTCAGCTGGCGGCC**TACAACATCGTAGGGCAGGC |
| C4S5_del_chk-FP | CAAACACAGAAGCTTTAACAACTCC |
| C4S6_del-FP | AGACCTTCTTTCGTGTAATACTACT |
| C4S6_del-RP | **CTGCAGCGTACGAAGCTTCAGCTGGCGGCC**AGCCTGACCCAAACTAACAACT |
| C4S6_del_chk-FP | TCCCGACTTCACTCCAGTAGA |
| C4S7_del-FP | CTCTTCCCAAAACCACTAGACTT |
| C4S7_del-RP | **CTGCAGCGTACGAAGCTTCAGCTGGCGGCC**ACACCGAGTAAAAAGCACCAT |
| C4S7_del_chk-FP | GCCAGTATTCTTGGACAACGT |
| C4S8_del-FP | TGCCGACTTGAGCCCTATTC |
| C4S8_del-RP | **CTGCAGCGTACGAAGCTTCAGCTGGCGGCC**ACGGTAAATCTCAACAGTCTCTT |
| C4S8_del_chk-FP | TGTGGTCTGCGCCTATTGTT |
| C4S8_del_chk1-FP | CACGTCACTTTCTTGCGCTC |
| C4S8_del_chk1-RP | TGTGCTGACATGATTCCGGA |
| C4S9_del-FP | AAGAACACGAGACAAGATGAGA |
| C4S9_del-RP | **CTGCAGCGTACGAAGCTTCAGCTGGCGGCC**CACCCCTGTTCTTGGTCCTG |
| C4S9_del_chk-FP | TCTCAACCACTGCATCTTCCA |
| C4S10_del-FP | AAACGTTAGACTGGCTGGGA |
| C4S10_del-RP | **CTGCAGCGTACGAAGCTTCAGCTGGCGGCC**TGTGAGGATCTATGCACATTTGT |
| C4S10_del_chk-FP | GCGCATTGCTAGAGAATGGC |
| C4S11_del-FP | AAGCGCGTCTGATGTTTACC |
| C4S11_del-RP | **CTGCAGCGTACGAAGCTTCAGCTGGCGGCC**AGCTCTACCCCTACTCAATGA |
| C4S11_del_chk-FP | CTGGCTGAAATGTCTCATGC |

* Sequences bolded and purple represent the 30 bp overlap used for overlap extension PCR throughout this study.

** Sequence bolded and blue represents the telomeric seed sequence.

*** Sequences highlighted in bold are homologous to the *kanMX* cassette used to select for transformants thoughout this study.

**** Primers labeled as C4Sx_del-FP or –RP were used to amplify the targeting sequence from genomic DNA isolated from the BYxRM F_2_ progenitor.

***** Primers labeled as C4Sx_del_chk-FP bind just upstream of the targeting sequence and were used to check for correct insertion of the deletion construct in conjunction with the pTEF-RP primer, which binds internally to the *kanMX* cassette and is depicted at the end of **Table S3**.

****** Primers labeled with ‘-HC’ in their name were used to ensure that one copy of the region of Chromosome IV targeted for deletion remained in successfully transformed aneuploid strains. This nomenclature is used in the following table as well.

**Table S4.** Primers used to make individual gene deletion constructs and check for proper placement, as well as for cloning *TSA2* into a low-copy *CEN6/ARS4* plasmid and checking *TSA2* copy number using qPCR.

| **Primer** | **Sequence(5'-3')** |
| --- | --- |
| TSA2_KO_up-FP * | TTCGGAGTATAAGCCCTGGG |
| TSA2_KO_up-RP | **CTGCAGCGTACGAAGCTTCAGCTGGCGGCC**CACGATAACGCTAGGCCCTA |
| TSA2_KO_down-RP ** | CACTATTACTGTTTTTTGCTCAAGAATATATTAGCCTTACAAGAACGTAAAAAACCAATC**CGCACTTAACTTCGCATCTG** |
| TSA2_KO-LC *** | ACCGACATAGTGAATGACTCCA |
| TSA2_KO-RC **** | GCGAGGCTCTCCTTTCTCTT |
| TSA2_KO-HC-FP | CGAAAACAAACCTGCATGGGA |
| TSA2_KO-HC-RP | TCCAGAGACTATGGCGTTTTGA |
| RPS18A_KO_up-FP | ACGGCAAGTGTTTCAATAGAGA |
| RPS18A_KO_up-RP | **CTGCAGCGTACGAAGCTTCAGCTGGCGGCC**CCGCTCTTGTGTATACGTTCA |
| RPS18A_KO_down-RP | GGAAGTGCTATGGAAATATATGTACTCGTACAAAAAAGATTAAGCTTGAATAATCTTATCAGT**CGCACTTAACTTCGCATCTG** |
| RPS18A_KO-LC | AAGCGTGTTCGGATTCCCAT |
| RPS18A_KO-RC | AAGGCAAGGAAAACCGCCTA |
| RPS18A_KO-HC-FP | AAGCGTGTTCGGATTCCCAT |
| RPS18A_KO-HC-RP | GCCGATTTTGTCATAGCTCACT |
| YDR455C_KO_up-FP | CGGGACATAACGCAAAGGTT |
| YDR455C_KO_up-RP | **CTGCAGCGTACGAAGCTTCAGCTGGCGGCC**AGAAAAGCAACGGAAACAGAAA |
| YDR455C_KO_down-RP | GAGAGAATGTATAAAGACTTAATTAATATATTTATATTAGAAACAAGGAAACCATACACTTTAAAGT**CGCACTTAACTTCGCATCTG** |
| YDR455C_KO-LC | TACCTGTACCACTTGGGCCA |
| YDR455C_KO-RC | TGAAAGCAGTCGGGTAACGT |
| YDR455C_KO-HC-FP | ATTGTAGCCTGCCGTGAGC |
| YDR455C_KO-HC-RP | GCTCTCTTGGCGGTGGTTAA |
| YHP1_KO_up-FP | CCAGCTTGGTTCTTGAACCG |
| YHP1_KO_up-RP | **CTGCAGCGTACGAAGCTTCAGCTGGCGGCC**AGCGGCCTGTTTCATAAATCT |
| YHP1_KO_down-RP | CCCTCGCCAGCCAGTGTAACTAGCATTTGTAGGGAAATAACAATAACACAAAATATCGTATATATATAAC**CGCACTTAACTTCGCATCTG** |
| YHP1_KO-LC | ATTGCAATAGCGGTCGGAGG |
| YHP1_KO-RC | GTGCAGGGATACGTCGGTAT |
| YHP1_KO-HC-FP | AGATTTATGAAACAGGCCGCT |
| YHP1_KO-HC-RP | AAGGCAAGGAAAACCGCCTA |
| TOM1_KO_up-FP | GGTGGTAATACTTTTGGAGGCC |
| TOM1_KO_up-RP | **CTGCAGCGTACGAAGCTTCAGCTGGCGGCC**CCTCATTATCACAAAGCGGCA |
| TOM1_KO_down-RP | AGACGTTCTAAAATACTTGGTTACATGGCGCTATAAATTTACACGAAAAATGA**CGCACTTAACTTCGCATCTG** |
| TOM1_KO-LC | AGAGGCTCCAAGGGCGATAA |
| TOM1_KO-RC | GCGAAATGCACATGAAATCTGT |
| TOM1_KO-HC-FP | CACGTTACCCGACTGCTTTC |
| TOM1_KO-HC-RP | CTGGATCGGTCCCACTCTTG |
| RPS17B_KO_up-FP | GGCGAAAATACATCATCCCCA |
| RPS17B_KO_up-RP | **CTGCAGCGTACGAAGCTTCAGCTGGCGGCC**TCATGTCGTCTGTTTTACTCCA |
| RPS17B_KO_down-RP | GCGTTGTATAACCTAGAGAAGAATAAATAGATAAAGAAAAAAGCAGATAAAA**CGCACTTAACTTCGCATCTG** |
| RPS17B_KO-LC | ACGCTTTCTTCCGTCACAGA |
| RPS17B_KO-RC | TTCATCTGGAAGACGCGCAT |
| RPS17B_KO-HC-FP | CGCTTCATAAAGTTGATCAAAGGTG |
| RPS17B_KO-HC-RP | CCGCTCAAAGAGACAGACGT |
| PPN1_KO_up-FP | AAGCGCGTCTGATGTTTACC |
| PPN1_KO_up-RP | **CTGCAGCGTACGAAGCTTCAGCTGGCGGCC**CACGATAACGCTAGGCCCTA |
| PPN1_KO_down-RP | AGAAACTGTAATTGAAGAATGATATGCATTTCTATGTGTATATTAAC**CGCACTTAACTTCGCATCTG** |
| PPN1_KO-LC | ACCGACATAGTGAATGACTCCA |
| PPN1_KO-RC | GCCACCATCAAACCTGACGT |
| PPN1_KO-HC-FP | ACCATTACCCGACTATTGAGGA |
| PPN1_KO-HC-RP | GGTCGACAGGATGGCAATCA |
| NHX1_partial_KO-FP *****/****** | CGCCATACCGGGCACCTTCATATCTGCTGTGGTTATTGGAATCATATTGTATA**CCTTGACAGTCTTGACGTGC** |
| NHX1_partial_KO-RP | GAGAGAATGTATAAAGACTTAATTAATATATTTATATTAGAAACAAGGAAACCATACACTTTAAAGT**CGCACTTAACTTCGCATCTG** |
| NHX1_partial_KO-LC | GATGTCCCCCGGGCATTATA |
| NHX1_partial_KO-RC | TGAAAGCAGTCGGGTAACGT |
| NHX1_partial_KO-HC-FP | GATGTCCCCCGGGCATTATA |
| NHX1_partial_KO-HC-RP | GTAACAGGGTCGGTAGCAGA |
| GUK1_KO-FP | GTCGCTTTGCGACAGCACTCTCCTACCAGGTACATACTTAGTAATGTCTATATAGGGCTACA**CCTTGACAGTCTTGACGTGC** |
| GUK1_KO-RP | CTACAGTTTACTTCGGATAAAGATTAGTAAACCTTTGCGTT**CGCACTTAACTTCGCATCTG** |
| GUK1_KO-LC | AACCAACAGATTCCTGGCCA |
| GUK1_KO-RC | ATGTGATGCGTCTTTCCGGC |
| GUK1_KO-HC-FP | AACCAACAGATTCCTGGCCA |
| GUK1_KO-HC-RP | CAACAAGAGGTTAAGCGCCG |
| UTP6_KO-FP | CTGATATGTAATTGCATTTATAAAATGTACAGTACCGCAT**CCTTGACAGTCTTGACGTGC** |
| UTP6_KO-RP | CTAAGGGCATAACATTGTGAAATTGACCAGAGATACACACATCACCGATTTCTAATA**CGCACTTAACTTCGCATCTG** |
| UTP6_KO-LC | GGCCAAGTCATAAAGGGACCTT |
| UTP6_KO-RC | TTTGTGCTTTTCCTGCGCTG |
| UTP6_KO-FP | GGCCAAGTCATAAAGGGACCTT |
| UTP6_KO-RP | TGAATGCAGAAGATGATCCTAGGT |
| ADA2_KO-FP | CACCCTCCATTTTCGATAAAATATCAGCGTAGTCTGAAAATATATACATTAAGCAAAAAGA**CCTTGACAGTCTTGACGTGC** |
| ADA2_KO-RP | GGTCCCTTTATGACTTGGCCAATAATAACTAGTGACAATTGTAGTTACTTTTCAATTTTTTTTTTG**CGCACTTAACTTCGCATCTG** |
| ADA2_KO-LC | CCGAATGTATGGATGTTACAGGG |
| ADA2_KO-RC | TGAATGCAGAAGATGATCCTAGGT |
| ADA2_KO-HC-FP | CCGAATGTATGGATGTTACAGGG |
| ADA2_KO-HC-RP | GGTAAGGGCGATGCTTTCCT |
| ECM11_KO-FP | AAATGTGCATAGATCCTCACATAGTATACAACTAAAAAGCAAACAAAAGAACATCCTCAA**CCTTGACAGTCTTGACGTGC** |
| ECM11_KO-RP | TAAGCATTTTGTTACTGAATATAGCACCTTTGATCAACTTTATGAAGCGT**CGCACTTAACTTCGCATCTG** |
| ECM11_KO-LC | ATTTTGGCACGGGTGATGTA |
| ECM11_KO-RC | CCGCTCAAAGAGACAGACGT |
| ECM11_KO-HC-FP | ATTTTGGCACGGGTGATGTA |
| ECM11_KO-HC-RP | TTATTTGGTCCTGAGCGGCT |
| pTEF-RP | GGGACAATTCAACGCGTCTG |
| tTEF-FP | CAGATGCGAAGTTAAGTGCG |
| TSA2_pRS410_Low_Copy-FP | CGCCGCTCGAGATACAAAACCATCTTTCTTATCATATATAGGG |
| TSA2_pRS410_Low_Copy-RP | TTTTCCTTTTGCGGCCGCCAATTAATATAGTGTAAAAGTTCTCAACGGGC |
| M13-FP | GTAAAACGACGGCCAGT |
| M13-RP | CAGGAAACAGCTATGAC |
| TSA2_qPCR-FP | CAGTCCACTGGAAACCTTCGA |
| TSA2_qPCR-RP | GTTCAAAAACAAGCCCCACCA |
| ACT1_qPCR-FP | GGTGTTCTTCTGGGGCAACT |
| ACT1_qPCR-RP | CGTCGGTAGACCAAGACACC |

* Primers labeled with ‘_up’ amplify a region just upstream of the targeted gene.

** Primers labeled with ‘_down’ amplify a region just downstream of the targeted gene and were used in conjunction with the Kan_overlap-FP depicted in **Table S2**.

*** Primers labeled with ‘-LC’ are homologous to regions just upstream of the targeting sequence and were used in conjunction with pTEF-RP to ensure correct placement of the deletion construct.

**** Primers labeled with ‘-RC’ are homologous to regions just downstream of the targeting sequence and were used in conjuction with tTEF-FP, which binds to the 3’ end of the amplified portion of the *kanMX* cassette.

***** Primers labeled with just ‘_KO-FP’ or ‘_KO-RP’ contain a 5’ tail with 30-60 bp of homology to the flanking regions of the targeted gene.

****** As *YDR455C* overlaps *NHX1*, in order to distinguish their individual effects, two separate deletions were constructed: one, labeled as ‘NHX1_partial’, was used to remove a region of *NHX1* ~270 bp upstream of the *YDR455C* translation start site, and the other, labeled as ‘YDR455C_KO’, was used to remove the coding regions of both genes.

**Note S1**. As mutants generated from the BYxRM and RMxYPS crosses had significantly higher gains in hydrogen peroxide tolerance compared to mutants from the BYxYPS cross, the progenitor and mutants from these crosses were sequenced first at high coverage. The BYxRM progenitor received approximately 150X coverage, while the RMxYPS progenitor received approximately 200X coverage. Mutants derived from BYxRM received an average of approximately 180X coverage, while mutants derived from RMxYPS received an average of approximately 220X coverage. After discovery of the Chromosome IV disomy in both crosses, the BYxYPS progenitor and mutants derived from it were sequenced at lower coverage to check for the presence of the duplication. The BYxYPS progenitor received approximately 7X coverage, while the mutants derived from it received an average of 17X coverage.

**Note S2**. The BYxRM aneuploid mutant that was used to generate the chromosome-scale deletion strains had additional point mutations in *GPB2*, a negative regulator of Ras-cAMP-PKA signalling, *SMP1*, an osmotic stress response transcription factor, and *YPRW delta14*, a Ty1 LTR. It is possible that one or more of these mutations may be at least partially responsible for the increased tolerance of some of the chromosome-scale deletion strains as compared to the euploid BYxRM progenitor.

**Note S3**. To confirm that duplication of *TSA2*had a causal role in increased tolerance in different strains bearing two copies of IV-R, knockouts were constructed in three additional strains. These included another fully disomic BYxRM mutant (Figure S7B), the partially disomic BYxRM mutant (Figure S7C), and a fully disomic RMxYPS mutant (Figure S7D). Deleting the coding region of *TSA2*in these additional IV-R mutants had a similar phenotypic effect to deleting *TSA2* in the BYxRM IV-R mutant used to originally map the effect of the chromosomal duplication (Figure S7A). However, while deleting *TSA2* in the other fully disomic BYxRM mutant (Figure S7B) did not fully recapitulate the globally calculated average MIC of the euploid BYxRM progenitor, it did fully recapitulate the average MIC of euploid progenitor strains phenotyped on the same plate as the other fully disomic BYxRM mutant (results not shown). This result suggests that, under certain conditions, *TSA2* may completely explain the increased tolerance of some of the aneuploid mutants obtained in our screen. It is also important to note that this particular BYxRM mutant had only a single detected point mutation in *YPRW delta14*, which, as it was detected in multiple independent BYxRM mutants, may be a false positive.

**Note S4**.  Identifying *TSA2* as the causal gene underlying the identified aneuploids allowed us to assess whether allelic differences among the BY, RM, and YPS strains at *TSA2* might explain why no aneuploids were observed in the BYxYPS cross. In such a case, the expectation would be that the RM allele would occur preferentially in the aneuploid strains. To check this possibility, we confirmed that the BYxRM progenitor strain carries the BY allele of *TSA2*, while the RMxYPS progenitor strain carries the YPS allele of *TSA2*. This finding implies that the allele state of *TSA2* does not explain the lack of aneuploidy in the BYxYPS cross.

**Note S5.** In the screen for increased tolerance on agar plates depicted in Figure 2A and B of the main text as well as Figure S4B, two disomic strains present in one of three technical replicates showed abnormal growth and were omitted as outliers from downstream analyses.
